# Reproductive factors and risk of lung cancer among 300,000 Chinese female never-smokers: evidence from the China Kadoorie Biobank study

**DOI:** 10.1186/s12885-024-12133-9

**Published:** 2024-03-26

**Authors:** Marwa M. A. Elbasheer, Bastian Bohrmann, Yiping Chen, Jun Lv, Dianjianyi Sun, Xia Wu, Xiaoming Yang, Daniel Avery, Liming Li, Zhengming Chen, Christiana Kartsonaki, Ka Hung Chan, Ling Yang

**Affiliations:** 1 Clinical Trial Service Unit and Epidemiological Studies Unit (CTSU), Nuffield Department of Population Health, University of Oxford, Oxford, UK; 2 Medical Research Council (MRC) Population Health Research Unit (MRC PHRU), University of Oxford, Oxford, UK; 3 Department of Epidemiology and Biostatistics, School of Public Health, Peking University, Beijing, China; 4 Center for Public Health and Epidemic Preparedness & Response, Peking University, Beijing, China; 5 Key Laboratory of Epidemiology of Major Diseases (Peking University), Ministry of Education, Beijing, China; 6 Pengzhou Centre for Disease Control and Prevention (CDC), Pengzhou, China; 7 Oxford BHF Centre of Research Excellence, University of Oxford, Oxford, UK

**Keywords:** Reproductive factors, Never-smokers, Lung cancer, Chinese females

## Abstract

**Background:**

Lung cancer is the leading cause of cancer mortality among Chinese females despite the low smoking prevalence among this population. This study assessed the roles of reproductive factors in lung cancer development among Chinese female never-smokers.

**Methods:**

The prospective China Kadoorie Biobank (CKB) recruited over 0.5 million Chinese adults (0.3 million females) from 10 geographical areas in China in 2004–2008 when information on socio-demographic/lifestyle/environmental factors, physical measurements, medical history, and reproductive history collected through interviewer-administered questionnaires. Cox proportional hazard regression was used to estimate adjusted hazard ratios (HRs) of lung cancer by reproductive factors. Subgroup analyses by menopausal status, birth year, and geographical region were performed.

**Results:**

During a median follow-up of 11 years, 2,284 incident lung cancers occurred among 282,558 female never-smokers. Ever oral contraceptive use was associated with a higher risk of lung cancer (HR = 1.16, 95% CI: 1.02–1.33) with a significant increasing trend associated with longer duration of use (p-trend = 0.03). Longer average breastfeeding duration per child was associated with a decreased risk (0.86, 0.78–0.95) for > 12 months compared with those who breastfed for 7–12 months. No statistically significant association was detected between other reproductive factors and lung cancer risk.

**Conclusion:**

Oral contraceptive use was associated with an increased risk of lung cancer in Chinese female never-smokers. Further studies are needed to assess lung cancer risk related to different types of oral contraceptives in similar populations.

**Supplementary Information:**

The online version contains supplementary material available at 10.1186/s12885-024-12133-9.

## Introduction

In China, lung cancer is the second most commonly diagnosed cancer among females and the leading cause of cancer-related mortality in both sexes [1]. The high incidence and mortality rates among Chinese males could be largely explained by the high prevalence of tobacco smoking among them [2, 3]. However, smoking is rare in Chinese females (< 5%) and is expected to reach 1.9% in 2025 [2–4]. Possible roles of environmental exposure to second-hand tobacco smoke, and household and outdoor air pollution have been suggested in studies in China [5–9]. However, these findings did not fully explain the relatively high risk of lung cancer among Chinese females.

The sex differences in lung cancer risk and prognosis are well documented with studies suggesting a higher risk of lung cancer among females at any given tobacco smoke exposure compared to males [10]; hence, questions have been raised on the possible role of oestrogen and other hormonal factors in lung cancer development and survival [11–13]. Existing evidence on the association of reproductive factors (as proxies of endogenous oestrogen exposure) with the risk of lung cancer has been inconclusive [14–17]. Similarly, inconsistency characterised the findings from observational studies on exogenous hormone exposure, including oral contraceptive (OC) and hormonal replacement therapy [18–23], with some reported variations by geographical region and smoking status [24, 25]. Findings from randomised control trials with a relatively small number of cases (< 200) suggested a higher risk of lung cancer mortality associated with hormonal therapy use but no association with the risk of lung cancer diagnosis [26, 27].

Most previous studies on reproductive factors and the risk of lung cancer among Chinese females were limited by small sample sizes (< 1000 participants), short follow-up duration (< 5 years), restricted to a single geographical region in the country or focused only on mortality [13, 28–30]. The present study utilised data from China Kadoorie Biobank (CKB), a population-based prospective cohort with > 300,000 Chinese females recruited from ten geographical regions in China, to assess the associations of reproductive characteristics with incident lung cancer risk among Chinese female never-smokers.

## Methods

### Study design and setting

The CKB is an ongoing population-based prospective cohort study, and details of the study design, methods and participant characteristics have been published previously [31, 32]. Briefly, the study recruited 512,715 Chinese adults (including 302,522 females) aged 30–79 years from five urban and five rural areas in China between June 2004 and July 2008. Extensive data collection was conducted at baseline via interviewer-administered questionnaires, which gathered information on socio-demographic, lifestyle and environmental factors (including duration, amount and frequency of tobacco smoking), medical history, and reproductive history for females. Trained staff collected blood samples and performed physical measurements (e.g. height, weight, lung function) following standardised protocols. Three resurveys were conducted in random samples of 4–5% of the surviving participants in 2008, 2013–2014, and 2020–2021. Ethical approvals were obtained from the Oxford Tropical Research Ethics Committee at the University of Oxford (Oxford, United Kingdom) and the Ethical Review Committee of the Chinese Centre for Disease Control and Prevention (CDC), Beijing, China. All participants provided written informed consent upon recruitment.

### Assessment of female reproductive factors

Self-reported information on female reproductive history was collected at baseline, including age at menarche, numbers of pregnancies, spontaneous or induced abortions, stillbirths and live births, age at birth and breastfeeding duration for each live birth, OC use, duration of use, OC starting age, menopausal status, age at menopause (for postmenopausal females), and history of hysterectomy and oophorectomy. For each post-menopausal female, the reproductive period was calculated as the duration between age at menarche and age at menopause.

### Follow-up and outcome definitions

Monitoring participants’ vital status was conducted by regularly screening official residential records and death certificates available from the regional CDC. Cancer incidence was ascertained through linkages to established cancer registries and national health insurance databases (covering ~ 98% of the study participants) using their unique national identification numbers. All events were coded by trained staff blinded to baseline data following the International Classification of Diseases, 10th Revision (ICD-10) [33]. The primary outcome of interest in the present study was incident lung cancer (ICD-10: C33-C34).

### Statistical analysis

Among 302,522 females recruited at baseline, after excluding ever smokers (defined as occasional smokers who had not completely stopped smoking for at least 6 months before baseline, those who had smoked ≥ 100 cigarettes but had quit smoking by choice for ≥ 6 months before baseline and regular smokers, *n* = 15,330); participants with a prior history of any cancer (*n* = 1,518); and participants who reported having been ever oral contraceptive users but reported 0 months as duration of use (*n* = 301), 285,373 females never-smokers (individuals who had smoked < 100 cigarettes during their lifetime [34]) remained. To reduce any influence of extreme values, females in the top and bottom 0.1% of age at menarche, age at first live birth and age at menopause were further excluded from the analysis (*n* = 2,171). In addition, the top 0.1% (*n* = 644) were excluded for the number of pregnancies and breastfeeding per child. There were no missing data in the remaining variables included in the analysis except for family history of any cancer (*n* = 10,098), which was assigned into a separate category in the subsequent analyses. Cohen’s kappa (κ) or Spearman correlations were used to assess the agreement between reported exposures at baseline and subsequent resurveys.

The categorisation of the exposure variables was performed in accordance with previous studies [35, 36], where appropriate, with some regrouping done based on the frequency distribution of specific variables as follows: age at menarche (< 13, 13–14, 15–16, > 16 years), number of pregnancies (never pregnant, 1–2, 3–4, > 4), parity defined as the total number of live births and stillbirths (nulliparous, 1, 2, 3–4, > 4 births), age at first birth (< 20, 20–22, 23- 25, > 25 years), average breastfeeding duration per child (never breastfed, < 7, 7–12, > 12 months), OC use (ever, never), duration of use (never users, ≤ 5 and > 5 years), age at starting using OC (never users, ≤ 25 and > 25 years old), menopausal status (pre- and perimenopausal, postmenopausal), age at menopause (< 43, 43–52, > 52 years), total reproductive period (< 30, 30- 35, > 35 years), history of oophorectomy (yes, no) and history of hysterectomy (yes, no).

Crude incidence rates of lung cancer were calculated by categories of each reproductive factor. Cox proportional hazard regression was used to estimate hazard ratios (HRs) and 95% confidence intervals (CIs) of incident lung cancer by each reproductive factor reported at baseline with age as the underlying time scale, and individuals were considered at risk from the age at study entry. The models were sequentially adjusted for the following covariates: age (years), study area (10 areas), occupation (agriculture and related workers, factory workers, administrative/technical/service workers, retired/unemployed, housewives, others), highest attained education (no formal schooling, primary school, middle school, high school, technical school/college/university), log-height and log-weight, physical activity calculated in metabolic equivalent of tasks (METs-h/day), family history of any cancer (yes, no, missing), personal history of lung disease including TB, COPD and asthma (yes/no) alcohol drinking (never/ever regular drinkers), frequency of environmental tobacco smoking exposure (never/almost never, < once/week, 1-2days/week, 3-5days/week, daily/almost daily), exposure to heating fuel during winter (no heating, coal, wood, central heating/gas/electricity/others) and cooking fuel (never/no cooking facility, coal, wood, gas/electricity/others). To assess for trend, categorical variables were entered into the model as numeric.

Additional analyses were conducted by further adjusting for age at menarche, the number of pregnancies, OC use, age at menopause, and history of oophorectomy or hysterectomy. The analysis of age at first birth and breastfeeding per child was conducted among parous females only (*n* = 279,107), while the analysis of age at menopause and reproductive period was restricted to postmenopausal females at the study baseline (*n* = 143,890). Breastfeeding was additionally grouped into three groups: never breastfed, ≤ 12 and > 12 months, to ensure comparability with subsequent subgroup analysis.

Subgroup analyses and likelihood ratio tests for interaction were conducted for child-bearing factors and OC use among subgroups defined by menopausal status, year of birth (before 1950/in or after 1950, a cut-off selected to investigate the impact of the one-child policy, assuming that females who were born after 1950 have reached childbearing age by the time of the policy implementation), and area (rural/urban). To maintain a sufficient number of cases in each category, never-pregnant and nulliparous females were excluded from the subgroup analysis, parity was regrouped into (1–2, 3–4, > 4 births), and average breastfeeding per child regrouped into (≤ 12, > 12months).

Sensitivity analyses were conducted by restricting the analysis to never-alcohol drinkers, never-oral contraceptive users, and those with no prior history of major lung diseases at baseline (TB, COPD and asthma). All analyses were performed using Stata/SE software version 16.1 (StataCorp, College Station, TX), and figures were plotted using R software version 3.3.2. We considered *p*-values < 0.05 as evidence of an association.

## Results

A total of 282,558 Chinese female never-smokers were included in this study. The median age of participants was 51 years (IQR: 42–58 years), and the majority were married (Table 1). Approximately 10% used OC, 50% were postmenopausal at baseline and had a median menopause age of 49 years (IQR: 46–51 years). Most of the study participants (97%) breastfed their babies, with a median duration of 12 months. In comparison to pre/perimenopausal females, postmenopausal females were more likely to be housewives, have no formal education, were less likely to be physically active or alcohol drinkers, had a higher median age at menarche, and higher averages of number of pregnancies and live births. In addition, postmenopausal females were more likely to have had an oophorectomy or hysterectomy, report having poor self-rated health, a family history of cancer and a personal prior history of lung disease.

**Table 1:** Baseline characteristics of the China Kadoorie Biobank female never-smokers by menopausal status

	**Pre/perimenopausal (*n* = 138,668)**	**Postmenopausal (*n* = 143,890)**	**Total (*n* = 282,558)**
Age, years – median (IQR)	42.2 (38.7—46.5)	57.9 (53.6—64.3)	50.5 (42.4–58.1)
Married	95.8%	84.0%	89.8%
Rural resident	58.3%	52.1%	55.1%
Housewives	7.8%	23.1%	15.6%
Have no formal schooling	13.4%	36.0%	24.9%
Weight, kg – mean ± SD	155.7 ± 5.7	152.9 ± 5.8	154.3 ± 5.9
Height, cm – mean ± SD	57.4 ± 8.9	56.2.7 ± 9.8	56.8 ± 9.4
Physical activity, (METs-h/d)^a^ – median (IQR)	22.1 (14.0—33.7)	13.4 (8.8—22.0)	16.9 (10.7–28.6)
Ever-alcohol drinker	39.8%	30.7%	35.2%
ETS exposure, daily/almost daily^a^	46.6%	37.3%	41.9%
Cooking fuel, clean energy^b^	44.3%	46.0%	45.2%
Heating fuel, clean energy^b^	20.8%	19.1%	19.9%
Age at menarche, years – mean ± SD	14.9 ± 1.7	15.9 ± 2.0	15.4 ± 1.9
No of pregnancies – median (IQR)	2 (2 -3)	4 (3 -5)	3 (2—4)
Parity – median (IQR)	1 (1—2)	3 (2—4)	2 (1—3)
Age at first live birth, years – median (IQR)	24 (22—25)	23 (20—25)	23 (21—25)
Ever breastfed	96.6%	97.9%	97.3%
Breastfeeding per child, months – median (IQR)	12 (10—18)	12 (12—19)	12 (11—18)
Ever-oral contraceptive use	9.7%	10.1%	9.9%
Duration of OC use, years, median (IQR)	2 (1—4)	2 (1—5)	2 (1—4)
OC use starting age, years, median (IQR)	25 (23—28)	27 (25—30)	26 (24—29)
Age at menopause, years – median (IQR)	-	49 (46—51)	49 (46—51)
Reproductive period, years – median (IQR)	-	33 (30—35)	33 (30—35)
Had oophorectomy	0.8%	2.1%	1.5%
Had hysterectomy	0.3%	7.3%	3.9%
Family history of cancer	15.5%	18.0%	16.8%
Poor self-rated health	8.3%	13.0%	10.7%
Personal history of lung disease^c^	4.5%	9.3%	6.9%

^a^*METs* (metabolic equivalent of tasks), *ETS* (environmental tobacco smoke exposure)

^b^Clean energy includes gas, electricity, central heating, others

^c^History of lung disease includes TB, COPD and asthma

Lung cancer cases were, on average, older, less likely to be physically active or married, more likely to live in rural areas, be housewives, have no formal education and report being postmenopausal at baseline (Supplementary Table 1). Compared to non-cases, they were also more likely to have been oral contraceptive users, to have had an ovary removed or a hysterectomy, to have poor self-rated health, a family history of cancer, or a personal history of lung disease.

Among 12,014 and 15,570 females interviewed in the first and second resurvey, respectively, agreement between the baseline and resurveys’ measures was high for most reproductive factors considered. For the binary variables, the agreement ranged from 87.4% to 98.3% and 72.8% to 97.2%, while for the continuous variables, the Spearman correlation coefficient range was 0.69 to 0.91 and 0.63 to 0.89 for the first and second resurveys, respectively. (Supplementary Table 2).

**Fig. 1:**
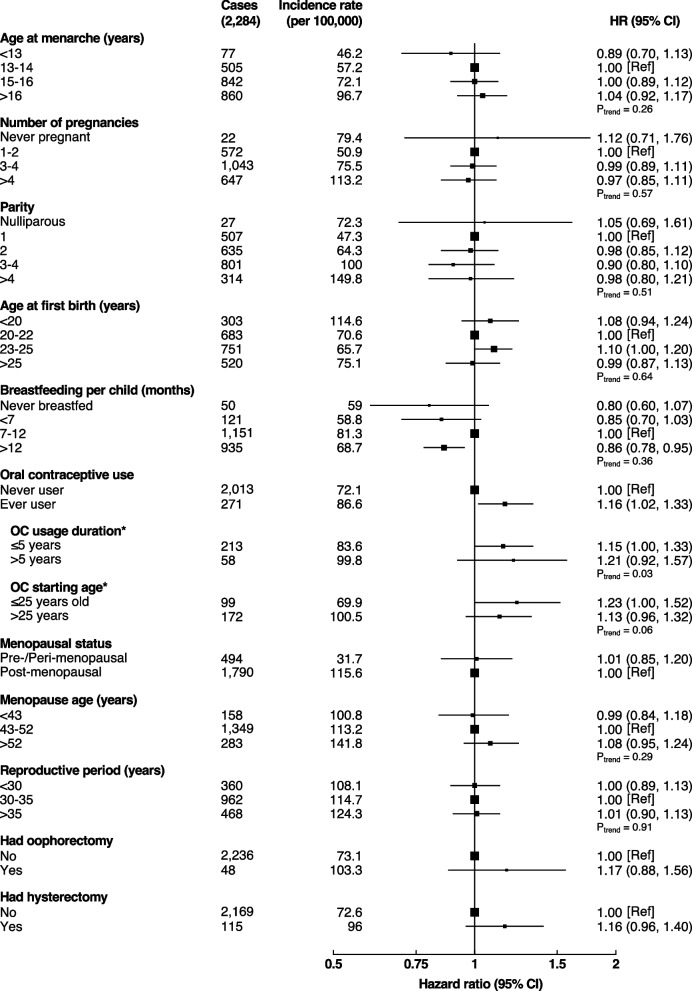
Relative risks of lung cancer associated with reproductive factors. Models were adjusted for age, study area, occupation, education, log-height, log-weight, physical activity, family history of any cancer, personal history of lung disease, alcohol drinking, frequency of environmental tobacco smoke exposure*,* heating fuel during winter and cooking fuel. *Relevent to never users

The median follow-up duration was 11 years (interquartile range [IQR]: 10–12 years), during which 2,284 (0.8%) developed lung cancer. Females who breastfed for more than one year per child had a lower risk of lung cancer (adjusted HR = 0.86, 95% CI: 0.78–0.95), compared to those who breastfed for 7–12 months; however, there was no evidence of a dose–response relationship with duration of breastfeeding (p- trend = 0.29), (Fig. 1). No substantial change in the relative risk was observed when > 12 months duration was compared to ≤ 12 months duration (Supplementary Table 3). Compared with never-users, females who ever used OCs had a significantly higher risk of lung cancer (1.16, 1.02–1.33) with a significant increasing trend associated with longer duration of use (p-trend = 0.03) and suggestive evidence of higher risk associated with starting OC usage at a younger age, (Fig. 1).

Overall, there was no statistical evidence of an association of age at menarche, number of pregnancies, parity, age at first birth, age at menopause, duration of reproductive period (all p-trend > 0.05) or menopausal status, oophorectomy, and hysterectomy with risk of lung cancer. No substantial changes in the results were observed after mutual adjustment for age at menarche, number of pregnancies, oral contraceptive use, age at menopause, and history of oophorectomy or hysterectomy (Supplementary Table 3).

**Fig. 2:**
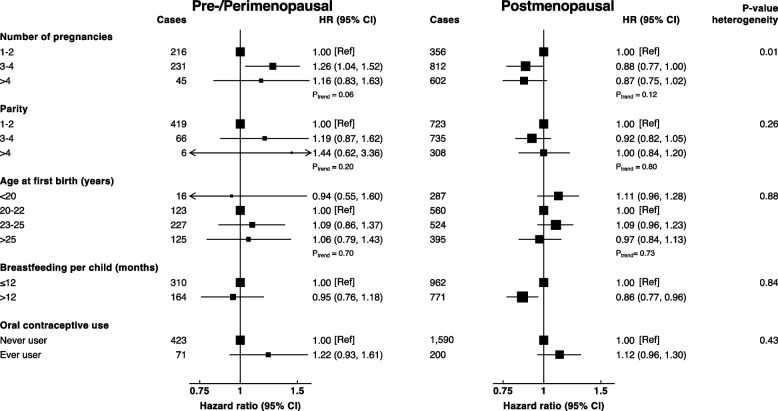
Adjusted hazard ratios of lung cancer according to childbearing factors by menopausal status. Models adjusted for age, region, occupation, education, log-height, log-weight, physical activity, family history of cancer, personal history of lung disease, alcohol drinking, frequency of environmental tobacco smoke exposure, heating fuel during winter, cooking fuel and oral contraceptive use

**Fig. 3:**
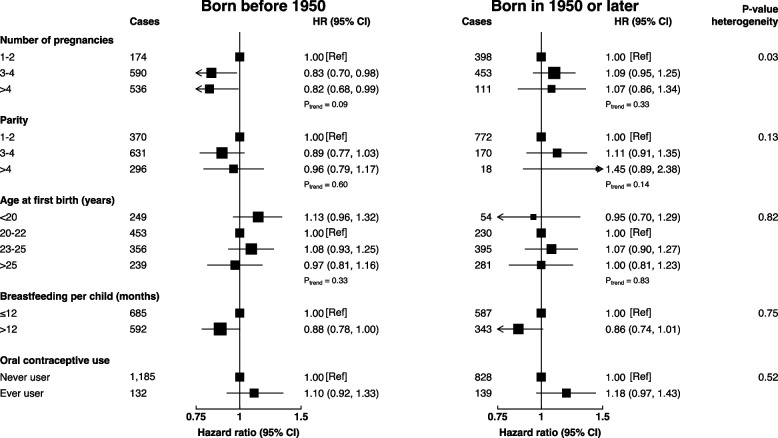
Adjusted hazard ratios of lung cancer according to childbearing factors by year of birth. Models adjusted for age, region, occupation, education, log-height, log-weight, physical activity, family history of cancer, personal history of lung disease, alcohol drinking, frequency of environmental tobacco smoke exposure, heating fuel during winter, cooking fuel and oral contraceptive use

In the subgroup analyses, there was some evidence that the association of the number of pregnancies with lung cancer may vary by menopausal status and year of birth (*p-interaction* = 0.01 and 0.03, respectively) (Figs. 2 and 3). The pattern of the association was positive among pre/perimenopausal females and those who were born in 1950 or later, and negative among their counterpart postmenopausal females and those who were born before 1950; however, no significant trend along with the number of pregnancies was observed among these groups. There was no evidence of effect modification of the remaining examined factors by menopausal status and year of birth.

**Fig. 4:**
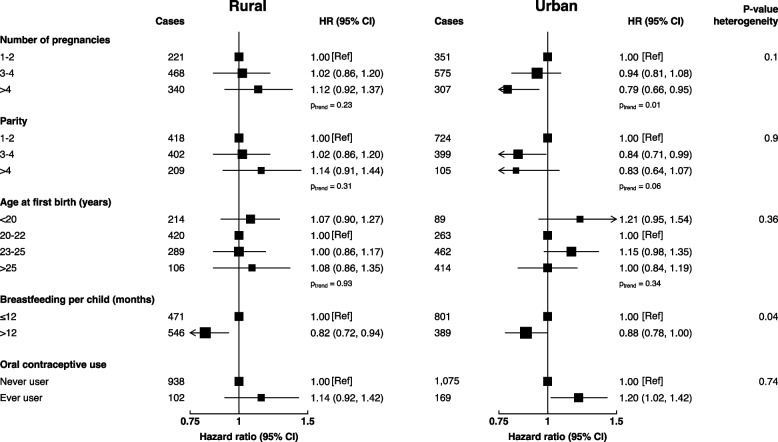
Adjusted hazard ratios of lung cancer according to childbearing factors by geographical regions. Models adjusted for age, region, occupation, education, log-height, log-weight, physical activity, family history of cancer, personal history of lung disease, alcohol drinking, frequency of environmental tobacco smoke exposure, heating fuel during winter, cooking fuel and oral contraceptive use

There was a statistically significant interaction between breastfeeding and geographical area in relation to lung cancer (*p-interaction* = 0.04), with lower risk of lung cancer for breastfeeding for > 12 months only significant in rural (0.82 [0.72—0.94]) but not urban residency (0.88 [0.78—1.00]) compared to those who breastfed for ≤ 12 months per child (Fig. 4). In addition, there was an inverse association of lung cancer with a higher number of pregnancies in urban but not rural areas (*p-trend* = 0.01).

There was no substantial change in the results of the associations of the number of pregnancies, parity, or age at first birth with risk of lung cancer after restricting the analysis to never-OC users, never-alcohol drinkers, and participants with no medical history of lung disease. However, there was some attenuation in the HR of lung cancer among OC users and the results were no longer significant after the exclusion of ever-alcohol drinkers. (Supplementary Table 4).

## Discussion

Among 282,558 Chinese female never-smokers, OC use was associated with a higher risk of lung cancer, while breastfeeding > 12 months was associated with a lower risk compared to breastfeeding for 7–12 months. In addition, the association of risk of lung cancer with the number of pregnancies varied by menopausal status and year of birth, while the risk from breastfeeding varied by geographical area.

Existing literature on the association of OC use with lung cancer was inconsistent. Prospective studies among 634,039 middle-aged females never smokers in the UK and 4,525,203 postmenopausal females in Korea found no evidence of association between the history or duration of oral contraceptive use and the risk of lung cancer [37, 38]. A pooled analysis of case–control and nested-case control studies from the International Lung Cancer Consortium showed a lower risk of lung cancer comparing former and current Asian OC users to never users [39]. In contrast, we found a higher risk associated with OC use among Chinese female never-smokers, with a significantly increasing trend associated with longer duration of use. These overall results were consistent with findings from a cohort of 107,171 postmenopausal nurses in the US [40], although the association was not statistically significant among our postmenopausal group, which might be due to its small size. A previous study on mortality among never-smokers in CKB (*n* = 814 lung cancer deaths) reported a significant trend of lung cancer mortality associated with duration of OC use with users for ≥ 10 years had almost twice the risk of developing lung cancer (1.97, 1.24–3.13) compared to never-users [13]. However, the latter study was limited by the very small number of cases among OC users (< 100). The attenuated and non significant HR of lung cancer among ever OC users in the sensitivity analysis after excluding ever alcohol drinkers may be due to the relatively small sample size after the exclusion of the latter group or residual confounding by alcohol.

Most CKB females breastfed their babies with a median duration of 12 months, and due to the very small number of women who had never breastfed, we could not assess the effect of ever breastfeeding versus never breastfeeding. We also categorised the variable into four categories with 7–12 group months as the reference to enable a direct investigation of the implication of 6 months of breastfeeding, a more common practice in Western populations. Numerous observational studies found no clear relationship between either history or duration of breastfeeding and lung cancer [15, 30, 41–43], but a cohort study of 42,615 Japanese female never-smokers showed an inverse association of ever breastfeeding with non-adenocarcinoma lung cancer (0.51, 0.28–0.92) compared to females who never breastfed [44]. Our study showed a lower risk associated with breastfeeding for more than one year compared to 7–12 months with no evidence of a dose–response relationship. No great difference in the relative risk among this group was observed when the risk was compared with those who breastfed for ≤ 12 months. This association appears to differ by geographical region, with a decreased risk in rural areas and no significant association in urban settings. A difference in reproductive factors, including average breastfeeding duration among our study population by area, has been documented previously, with females in rural areas being more likely to breastfeed their babies and for a longer duration on average compared to their counterparts in urban areas [36]. These geographical differences might have contributed to the variation by area and resulted in a stronger association of breastfeeding with lung cancer in rural regions in the present study. However, a possible role of chance could not be ruled out.

Regarding other reproductive factors, two large prospective cohort studies among females in the UK and South Korea reported no association between age at menarche, age at menopause, age at first birth, number of pregnancies, parity and reproductive period and the risk of lung cancer [37, 38], in consistency with our findings. On the other hand, higher and lower risk of lung cancer associated with childbearing factors, reproductive period, age at menopause, and type of menopause has been observed by studies among Asian populations and elsewhere [16–18, 28–30, 40, 44, 45]. This inconsistency in the findings across studies could be attributed to many factors including different study designs, the limited sample size of many studies, residual confounding by age and smoking, and lack of adjustment for important confounders in some studies such as smoking, environmental tobacco smoke exposure, and socioeconomic factors. In addition to the differences in OC use and the underlying variation between populations in factors such as breastfeeding habits and the classification of what is considered for instance as early or late age at menarche and age at menopause, and the contribution of these variations to the overall association.

Interestingly, the association of number of pregnancies with lung cancer varied by geographical region, year of birth and menopausal status. An inverse association was found in urban residents, among those who were born before 1950 and postmenopausal females, while no significant association was found in their respective counterparts. These variations might be explained by biological changes such as lower oestrogen levels due to menopause. Our findings are in line with a pooled analysis of case–control studies that reported variation in the association between parity, which is highly correlated with number of pregnancies, and risk of lung cancer by menopausal status with premenopausal females having increased risk associated with parity while no association among peri/postmenopausal females [15].

On the other hand, as the females born in or after the 1950s reached childbearing age by the 1970s or later, the heterogeneity in lung cancer risk by year of birth might be explained by the introduction of the one-child policy in the late 1970s, other demographic and socio-economic factors including the great famines in 1958–61, economic development, and urbanisation during this time period [46]. In general, with significant regional variations, these political and socioeconomic changes resulted in striking changes in the reproductive characteristics and patterns among Chinese females such as a decrease in mean age at menarche, an increase in mean age at first birth, a sudden increase in spacing between births and a steady decline in parity since the 1950s [36, 46]. All this in return, might have contributed to the variations between the two birth cohorts in the association of number of pregnancies with lung cancer.

Experimental studies on animals showed that female mice are more sensitive to chemical carcinogens of the lung and that lung cancer development among females could be inhibited by ovariectomy, suggesting that sex hormones may play a role in lung carcinogenesis [10]. Furthermore, studies have shown that premenopausal females are more likely to have more advanced disease at presentation and poor survival than postmenopausal females [47, 48]. Findings also showed the expression of oestrogen receptor β in normal and cancerous lung tissues and cell lines, suggesting the responsiveness of lung tissue to oestrogen stimulation [49, 50]. Overall, our findings supported the hypothesis regarding the potential involvement of sex hormones -particularly oestrogen- in lung carcinogenesis, as reflected by the increased risk associated with OC use, lower risks associated with a higher number of pregnancies among postmenopausal females or females who breastfed their children for a longer duration, compared with their counterparts. Breastfeeding down-regulates oestradiol concentrations with lactating females having considerably lower levels compared to non-lactating females [51]. In addition, a low level of circulating oestrogen metabolites among parous females compared to nulliparous has been reported previously [52]. The level of these metabolites was positively associated with time since last birth suggesting that giving birth may enhance the down-regulation of ovarian function and hence reduce cumulative exposure to free estradiol [52].

The strengths of this study include its prospective design, large sample size, long follow-up duration, and the detailed assessment of medical history, and lifestyle and environmental factors that allowed taking into account important confounders. In addition to the wide range and the good reliability of self-reported reproductive factors, which allowed accurate estimation of associations with risk of lung cancer among female never-smokers.

There are also some limitations in the present study. First, despite the large sample size, the number of cases was small in some subgroups, thus resulting in low statistical power to detect modest associations (if any) in some analyses. Second, the study did not assess the associations between reproductive factors and the risk of different subtypes of lung cancer due to the current lack of complete histopathological data. Future studies on risk stratification by tumour subtype are recommended when the data become available. Third, the study also did not account for changes in menopausal status during follow-up as some perimenopausal females may have reached menopause by the time of the development of the disease, and neither did it account for the type of OC and the changes in usage among premenopausal females. Finally, the role of chance in some of the results cannot be ruled out and although major risk factors were adjusted, residual confounding may still exist.

In conclusion, based on this large Chinese female never smokers cohort, the present study showed a decreased risk of lung cancer associated with breastfeeding for a longer duration while an increased risk with OC use. Future studies are needed to assess the association of OC type with each tumour subtype, as well as to understand the potential mechanisms underlying the observed associations.

### Supplementary Information

**Additional file 1. **Baseline characteristics of the China Kadoorie Biobank female never-smokers by lung cancer status.

**Additional file 2. **Agreement between baseline reproductive factors and subsequent resurveys.

**Additional file 3. **Relative risks of lung cancer associated with reproductive factors after mutual adjustment for reproductive factors.

**Additional file 4. **Adjusted hazard ratios of lung cancer after restricting the analysis to never-alcohol drinkers, never-oral contraceptive users, and participants without a history of lung disease.

## Data Availability

The dataset used and/or analysed during the current study is available from the corresponding author on reasonable request. Further information on China Kadoorie Biobank data access policies and procedures is available at: https://www.ckbiobank.org/data-access
