# Supplementary material for: Reproductive factors and risk of lung cancer among 300,000 Chinese female never-smokers: evidence from the China Kadoorie Biobank study
Source: BMC Cancer. 2024 Mar 26;24:384. doi: 10.1186/s12885-024-12133-9 (PMC10964706; doi:10.1186/s12885-024-12133-9)
Supplement: Supplementary file 1 — Additional file 1. Baseline characteristics of the China Kadoorie Biobank female never-smokers by lung cancer status. [file 12885_2024_12133_MOESM1_ESM.docx]

**Supplementary 1. Baseline characteristics of the China Kadoorie Biobank female never-smokers by lung cancer status**

|  | **Had lung cancer (2,284)** | **Had no lung cancer (280,274)** | **Total (282,558)** |
| --- | --- | --- | --- |
| Age, years - median (IQR) | 58.8 (51.7-66.2) | 50.4 (42.3-58.0) | 50.5 (42.4-58.1) |
| Married | 84.4% | 89.8% | 89.8% |
| Rural resident | 45.5% | 55.2% | 55.1% |
| Housewives | 21.8 | 15.6% | 15.6% |
| Have no formal schooling | 32.4% | 24.8% | 24.9% |
| Weight, kg - mean±SD | 56.3 ± 9.8 | 56.8 ± 9.4 | 56.8 ± 9.4 |
| Height, cm - mean±SD | 153.6± 5.9 | 154.3± 5.9 | 154.3± 5.9 |
| Physical activity, (METs-h/d) - median (IQR) | 13.0 (8.4-20.9) | 17.0 (10.8-28.6) | 16.9 (10.7-28.6) |
| Ever-alcohol drinker | 29.8% | 35.2% | 35.2% |
| ETS exposure, daily/almost daily* | 35.2% | 41.9% | 41.9% |
| Cooking fuel, clean energy† | 53.0% | 45.1% | 45.2% |
| Heating fuel, clean energy† | 24.2% | 19.9% | 19.9% |
| Age at menarche, years - mean±SD | 15.9 ± 2.0 | 15.4 ± 1.9 | 15.4 ± 1.9 |
| No of pregnancies - median (IQR) | 4 (2 - 5) | 3 (2 - 4) | 3 (2 - 4) |
| Parity - median (IQR) | 2(2 - 4) | 2 (1 - 3) | 2 (1 - 3) |
| Age at firs live birth, years - median (IQR) | 23 (21 - 25) | 23 (21 - 25) | 23 (21 - 25) |
| Ever breastfed | 97.8% | 97.2% | 97.3% |
| Breastfeeding per child, months - median (IQR) | 12 (11 - 18) | 12 (11 - 18) | 12 (11 - 18) |
| Ever-oral contraceptive use | 11.9% | 9.9% | 9.9% |
| Duration of OC use, years, median (IQR) | 2 (1 - 5) | 2 (1 - 4) | 2 (1 - 4) |
| OC use starting age, years, median (IQR) | 27 (25 - 30) | 26 (24 - 29) | 26 (24 - 29) |
| Postmenopausal | 78.4% | 50.7% | 50.9% |
| Age at menopause, years | 49 (46 - 51) | 49 (46 - 51) | 49 (46 - 51) |
| Reproductive period, years | 33 (30 - 36) | 33 (30 - 35) | 33 (30 - 35) |
| Had ovary removed | 2.1% | 1.5% | 1.5% |
| Had hysterectomy | 5.0% | 3.9% | 3.9% |
| Family history of cancer | 19.0% | 16.7% | 16.8% |
| Poor self-rated health | 13.1% | 10.7% | 10.7% |
| Personal history of lung disease | 10.6% | 6.9% | 6.9% |

*METs (metabolic equivalent of tasks), ETS (environmental tobacco smoke exposure)

**†**Clean energy includes gas, electricity, central heating, others

**ǂ**History of lung disease includes TB, COPD and asthma
