# Supplementary material for: Reproductive factors and risk of lung cancer among 300,000 Chinese female never-smokers: evidence from the China Kadoorie Biobank study
Source: BMC Cancer. 2024 Mar 26;24:384. doi: 10.1186/s12885-024-12133-9 (PMC10964706; doi:10.1186/s12885-024-12133-9)
Supplement: Supplementary file 2 — Additional file 2. Agreement between baseline reproductive factors and subsequent resurveys. [file 12885_2024_12133_MOESM2_ESM.docx]

**Supplementary 2.** **Agreement between baseline reproductive factors and subsequent resurveys**

|  | **First resurvey (n=12,014)** | | **Second resurvey (n=15,570)** | |
| --- | --- | --- | --- | --- |
|  | **Agreement*** | **Kappa/Spearman Coefficient†** | **Agreement** | **Kappa/Spearman Coefficient** |
| Age at menarche, years | - | 0.85 | - | 0.81 |
| No. of pregnancies | - | 0.89 | - | 0.85 |
| Age at first birth, years | - | 0.91 | - | 0.89 |
| Breastfeeding duration for 1^st^ child, months | - | 0.69 | - | 0.63 |
| Menopausal status | 87.4% | 0.76 | 72.8% | 0.47 |
| Age at menopause, years | - | 0.80 | - | 0.73 |
| Oral contraceptive use | 94.3% | 0.66 | 92.6% | 0.58 |
| Duration of OC use, years | - | 0.68 | - | 0.58 |
| OC use starting age, years | - | 0.75 | - | 0.63 |
| Had oophorectomy | 98.3% | 0.46 | 97.2% | 0.36 |
| Had hysterectomy | 98.0% | 0.76 | 96.2% | 0.64 |

*Represents the proportion of participants who reported the same response at baseline and subsequent resurveys.

**†** Cohen’s kappa (κ) for binary variables and Spearman coefficient for continuous variables.
