# Supplementary material for: Reproductive factors and risk of lung cancer among 300,000 Chinese female never-smokers: evidence from the China Kadoorie Biobank study
Source: BMC Cancer. 2024 Mar 26;24:384. doi: 10.1186/s12885-024-12133-9 (PMC10964706; doi:10.1186/s12885-024-12133-9)
Supplement: Supplementary file 3 — Additional file 3. Relative risks of lung cancer associated with reproductive factors after mutual adjustment for reproductive factors. [file 12885_2024_12133_MOESM3_ESM.docx]

**Supplementary 3. Relative risks of lung cancer associated with reproductive factors after mutual adjustment for reproductive factors**

|  | **Had lung cancer (2,284)** | **HR (95%CI)** | **P- trend** |
| --- | --- | --- | --- |
| **Age at menarche, years** |  |  |  |
| ≤12 | 77 | 0.89 (0.70 - 1.14) | 0.23 |
| 13-14 | 505 | 1 |  |
| 15-16 | 842 | 1.00 (0.89 - 1.12) |  |
| ≥17 | 860 | 1.04 (0.93 - 1.17) |  |
| **No of pregnancies** |  |  |  |
| Never pregnant | 22 | 1.12 (0.71 - 1.77) | 0.44 |
| 1-2 | 572 | 1 |  |
| 3-4 | 1,043 | 0.98 (0.88 - 1.10) |  |
| ≥5 | 647 | 0.96 (0.84 - 1.10) |  |
| **Parity** |  |  |  |
| Nulliparous | 27 | 1.06 (0.69 - 1.63) | 0.52 |
| 1 | 507 | 1 |  |
| 2 | 635 | 0.97 (0.85 - 1.11) |  |
| 3-4 | 801 | 0.92 (0.78 - 1.08) |  |
| ≥5 | 314 | 0.98 (0.80 - 1.21) |  |
| **Age at 1st birth*, years** |  |  | 0.93 |
| ≤19 | 303 | 1.09 (0.95 - 1.26) |  |
| 20-22 | 683 | 1 |  |
| 23-25 | 751 | 1.09 (0.98 - 1.22) |  |
| ≥26 | 520 | 0.99 (0.87 - 1.13 |  |
| **Breastfeeding per child*, months** |  |  |  |
| Never breastfed | 50 | 0.80 (0.60 - 1.07) | 0.90 |
| ≤6 | 121 | 0.85 (0.70 - 1.03) |  |
| 7-12 | 1,151 | 1 |  |
| > 12 | 935 | 0.85 (0.77 - 0.94) |  |
| **Breastfeeding per child*, months** |  |  |  |
| Never breastfed | 50 | 0.82 (0.62 - 1.09) | 0.04 |
| ≤12 | 1,320 | 1 |  |
| >12 | 887 | 0.87 (0.79 - 0.96) |  |
| **Oral contraceptive (OC) use** |  |  |  |
| Never user | 2,013 | 1 | - |
| Ever user | 271 | 1.17 (1.02 - 1.34) |  |
| ≤5 years | 213 | 1.16 (1.00 - 1.34) | 0.02 |
| >5 years | 58 | 1.21 (0.93 - 1.58) |  |
| OC use starting age, years |  |  |  |
| ≤25 years | 99 | 1.24 (1.00 - 1.53) | 0.05 |
| >25 years | 172 | 1.13 (0.96 - 1.33) |  |
| **Menopausal status** |  |  |  |
| Pre-/perimenopause | 494 | 1.03 (0.87 - 1.23) | - |
| Post-menopause | 1,790 | 1 |  |
| **Menopause age, years** |  |  |  |
| ≤42 | 158 | 0.98 (0.82 - 1.16) | 0.22 |
| 43-52 | 1,349 | 1 |  |
| ≥53 | 283 | 1.09 (0.95 - 1.24) |  |
| **Reproductive period, years** |  |  |  |
| ≤29 | 360 | 0.99 (0.87 - 1.12) | 0.75 |
| 30-35 | 962 | 1 |  |
| ≥36 | 468 | 1.01 (0.91 - 1.13) |  |
| **Had oophorectomy** |  |  |  |
| No | 2,236 | 1 | - |
| Yes | 48 | 1.10 (0.82 - 1.50) |  |
| **Had hysterectomy** |  |  |  |
| No | 2,169 | 1 | - |
| Yes | 115 | 1.15 (0.94 - 1.42) |  |

*Among parous females only
