# Supplementary material for: Reproductive factors and risk of lung cancer among 300,000 Chinese female never-smokers: evidence from the China Kadoorie Biobank study
Source: BMC Cancer. 2024 Mar 26;24:384. doi: 10.1186/s12885-024-12133-9 (PMC10964706; doi:10.1186/s12885-024-12133-9)
Supplement: Supplementary file 4 — Additional file 4. Adjusted hazard ratios of lung cancer after restricting the analysis to never-alcohol drinkers, never-oral contraceptive users, and participants without a history of lung disease. [file 12885_2024_12133_MOESM4_ESM.docx]

**Supplementary 4.** Adjusted hazard ratios of lung cancer after restricting the analysis to never-alcohol drinkers, never-oral contraceptive users, and participants without a history of lung disease

|  | **Never alcohol drinkers** | | | **Never oral contraceptive users** | | | **Participants with no history of lung disease** | | |
| --- | --- | --- | --- | --- | --- | --- | --- | --- | --- |
|  | **Cases** | **HR (95%CI)** | **p-trend** | **Cases** | **HR (95%CI)** | **p-trend** | **Cases** | **HR (95%CI)** | **p-trend** |
| **No of pregnancies** |  |  |  |  |  |  |  |  |  |
| Never pregnant | 16 | 1.14 (0.67 - 1.96) | 0.52 | 22 | 1.15 (0.73 - 1.83) | 0.37 | 18 | 1.15 (0.69 - 1.90) | 0.92 |
| 1-2 pregnancies | 390 | 1.00 [Ref] |  | 518 | 1.00 [Ref] |  | 517 | 1.00 [Ref] |  |
| 3-4 pregnancies | 732 | 0.98 (0.86 - 1.12) |  | 909 | 0.98 (0.87 - 1.10) |  | 942 | 1.03 (0.92 - 1.15) |  |
| ≥5 pregnancies | 465 | 0.96 (0.82 - 1.13) |  | 564 | 0.95 (0.82 - 1.10) |  | 564 | 1.02 (0.88 - 1.17) |  |
| **Parity** |  |  |  |  |  |  |  |  |  |
| Nulliparous | 19 | 1.09 (0.65 - 1.81) | 0.76 | 27 | 1.11 (0.72 - 1.71) | 0.35 | 23 | 1.12 (0.71 - 1.78) | 0.77 |
| 1 | 295 | 1.00 [Ref] |  | 433 | 1.00 [Ref] |  | 469 | 1.00 [Ref] |  |
| 2 | 454 | 1.01 (0.86 - 1.19) |  | 541 | 0.98 (0.85 - 1.14) |  | 567 | 0.98 (0.85 - 1.13) |  |
| 3-4 | 588 | 0.94 (0.77 - 1.13) |  | 719 | 0.90 (0.76 - 1.07) |  | 724 | 0.97 (0.82 - 1.15) |  |
| ≥5 | 247 | 1.03 (0.80 - 1.31) |  | 293 | 0.96 (0.77 - 1.20) |  | 258 | 1.00 (0.79 - 1.25) |  |
| **Age at first birth** |  |  |  |  |  |  |  |  |  |
| ≤19 | 254 | 1.10 (0.94 - 1.29) | 0.35 | 272 | 1.07 (0.92 - 1.24) | 0.86 | 257 | 1.06 (0.92 - 1.24) | 0.92 |
| 20-22 | 520 | 1.00 [Ref] |  | 606 | 1.00 [Ref] |  | 596 | 1.00 [Ref] |  |
| 23-25 | 493 | 1.05 (0.92 - 1.20) |  | 645 | 1.09 (0.97 - 1.23) |  | 690 | 1.12 (0.99 - 1.26) |  |
| ≥26 | 317 | 0.97 (0.82 - 1.13) |  | 463 | 1.01 (0.87 - 1.16) |  | 475 | 1.00 (0.87 - 1.15) |  |
| **Breastfeeding per child** |  |  |  |  |  |  |  |  |  |
| Never breastfed | 30 | 0.78 (0.54 - 1.13) | 0.21 | 45 | 0.80 (0.59 - 1.08) | 0.70 | 42 | 0.75 (0.55 - 1.03) | 0.41 |
| <7 | 85 | 0.91 (0.73 - 1.14) |  | 92 | 0.74 (0.60 - 0.92) |  | 107 | 0.84 (0.69 - 1.03) |  |
| 7-12 | 849 | 1.00 [Ref] |  | 996 | 1.00 [Ref] |  | 1,047 | 1.00 [Ref] |  |
| >12 | 620 | 0.85 (0.76 - 0.95) |  | 853 | 0.85 (0.77 - 0.95) |  | 822 | 0.85 (0.76 - 0.94) |  |
| **Oral contraceptive use** |  |  |  |  |  |  |  |  |  |
| Never user | 1,421 | 1.00 [Ref] | - | - | - | - | 1,792 | 1.00 [Ref] | - |
| Ever user | 182 | 1.11 (0.94 - 1.31) |  | - | - |  | 249 | 1.18 (1.02 - 1.35) |  |
